# Physiological parameters of mental health predict the emergence of post-traumatic stress symptoms in physicians treating COVID-19 patients

**DOI:** 10.1038/s41398-021-01299-6

**Published:** 2021-03-15

**Authors:** T. Dolev, S. Zubedat, Z. Brand, B. Bloch, E. Mader, O. Blondheim, A. Avital

**Affiliations:** 1 Technion—Israel Institute of Technology, Faculty of Medicine, Department of Neuroscience, Haifa, Israel; 2 Emek Medical Center, Department of Psychiatry, Afula, Israel

**Keywords:** Scientific community, Biomarkers

## Abstract

Lack of established knowledge and treatment strategies, and change in work environment, may altogether critically affect the mental health and functioning of physicians treating COVID-19 patients. Thus, we examined whether treating COVID-19 patients affect the physicians’ mental health differently compared with physicians treating non-COVID-19 patients. In this cohort study, an association was blindly computed between physiologically measured anxiety and attention vigilance (collected from 1 May 2014 to 31 May 31 2016) and self-reports of anxiety, mental health aspects, and sleep quality (collected from 20 April to 30 June 2020, and analyzed from 1 July to 1 September 2020), of 91 physicians treating COVID-19 or non-COVID-19 patients. As a priori hypothesized, physicians treating COVID-19 patients showed a relative elevation in both physiological measures of anxiety (95% CI: 2317.69–2453.44 versus 1982.32–2068.46; *P* < 0.001) and attention vigilance (95% CI: 29.85–34.97 versus 22.84–26.61; *P* < 0.001), compared with their colleagues treating non-COVID-19 patients. At least 3 months into the pandemic, physicians treating COVID-19 patients reported high anxiety and low quality of sleep. Machine learning showed clustering to the COVID-19 and non-COVID-19 subgroups with a high correlation mainly between physiological and self-reported anxiety, and between physiologically measured anxiety and sleep duration. To conclude, the pattern of attention vigilance, heightened anxiety, and reduced sleep quality findings point the need for mental intervention aimed at those physicians susceptible to develop post-traumatic stress symptoms, owing to the consequences of fighting at the forefront of the COVID-19 pandemic.

## Introduction

First identified in December 2019, the SARS-CoV-2 COronaVIrus Disease-19 (COVID-19) rapidly became a global pandemic by March 2020. Currently, there are over 50 million reported confirmed cases in >190 countries with a 3% death rate^1^. In Israel, similar to the US and many European countries, the rise in infectious rate to several thousand (relative to 9 M population) per day has led to lockdown and the opening of 3–5 COVID-19 wards in each public hospital.

Prior to the COVID-19 pandemic, physicians’ work hours were already a topic of growing worldwide debate^2^. Given the rapid acceleration of transmission combined with uncertainty and lack of knowledge and treatment strategies, the pandemic has been imposing an even greater workload among frontline medical services, exposing starved public health systems on the verge of collapse.

An indication for higher psychological stress among the medical staff during the outbreak of COVID-19 has been accumulating^3^, indicating that stress of frontline staff, as well as the number of working hours per week, correlate with perceived anxiety^4^. Accordingly, medical staff in China who treated COVID-19 patients during January and February 2020 had elevated anxiety and stress-dependent on their reduced sleep quality^5^.

The lack of proper sleep results in psychological distress, influences behavioral performance^6,7^, and impairs attention and working memory^8^. As previously shown by Landrigan et al.^9^, medical residents made substantially more serious medical errors under a frequent 24-hour shift schedule, manifesting significantly more polysomnographically-recorded attentional failures during work hours^10^.

Sleep is related to a neurological sensorimotor physiologic phenomenon called pre-pulse inhibition (PPI), which is the ability to inhibit the response to a startling acoustic pulse by the preceded pre-pulse weak stimulus. Sleep deprivation is known to disrupt this reflex, generating cognitive failures, and affecting the startle response^11,12^. It was recently suggested by our research group^13^ and others^14^ that PPI is modulated by attentional functioning. Hence, sleep deprivation might have a great cognitive (attention) and emotional (anxiety) cost that may directly affect clinical decision-making and performance.

The emergent workload imposed by the COVID-19 outbreak has emphasized the need to identify physicians at risk of burnout under the stressful conditions of the pandemic. Common tools (e.g., questionnaire) aiming to understand and predict physicians’ burnout are of limited scope, both in objectivity and face validity (e.g., focusing on surgical trainees)^15^, thereby compromising their utility. It is not clear whether any single measure can consistently and reliably describe the multidimensionality of working in a highly demanding profession, particularly during the unique circumstances of a pandemic. Thus, adapting machine learning (ML) is suggested to provide more nuanced insights into the physicians’ wellness. Previous reports have suggested that the use of ML techniques in trauma-related disorders is extensive; clustering techniques have been used to define outcome profiles^16^ and lead to improved characterization of a disease phenotype^17,18^. Thus, ML methods may be used in forecasting post-traumatic stress disorder (PTSD)^19^. Therefore, we aim to assess whether a pre-COVID-19 physiologically measured attention vigilance and anxiety (before and after a 24-hrs shift challenge) can serve as predictors of self-reported anxiety, sleep quality, and mental consequences (i.e., psychological, emotional, and social) while treating COVID-19 patients.

## Subjects and methods

### Subjects

The study included 105 medical residents in the screening phase (2014–2016), out of which 91 re-participated as senior physicians in the test phase (2020). Both sample sizes meet power calculation of >85%. Participants in the screening phase anonymously volunteered and were excluded according to the following criteria: a diagnosed attention deficit disorder or sleep disorder, hearing deficit, active psychiatric or neurological disease, jet lag, regular use of sedative or stimulant prescription, and pregnancy or breast-feeding.

The screening phase was conducted in the Emek Medical Center (Israel), a large academic hospital located in the north of Israel. For anonymity and safety reasons during the test phase, questionnaires were sent to an unspecified e-mail list as created in the screening phase. Age, sex, department, and daily caffeine and cigarette consumption were documented. Subjects who completed the questionnaires (*n* = 91) were then categorized into two groups to allow a blinded analysis of the study outcomes. The identity of COVID-19 or non-COVID-19 groups was revealed after completing all analyses.

### Procedure

#### Screening phase

In total, 105 medical residents were physiologically measured for attention vigilance (auditory sustained attention test) and anxiety (startle response) at baseline (08:00—10:00AM) and following a 24-hrs shift challenge. Subjects reported normal sleep duration the night before the study as well as a normal shift workload. Consumption of caffeine and nicotine was controlled to address possible confounding effects^20–22^. We initially planned this study to be a follow-up study, examining the physiological measures every 5 years, to detect the burnout trajectory of physician during their routine workload. However, as the unique outbreak of the COVID-19 pandemic, we decided to shift from our original plan.

#### Test phase

Four years later (April–June 2020), for safety reasons, questionnaires (Hebrew editions) were sent to the physicians using an unspecified e-mail list of those who participated in the screening phase. Upon completion, the questionnaires were sent back anonymously to the research office and coded as group 1 or 2 (COVID-19 vs non-COVID-19, respectively). Ninety-seven out of 105 physicians re-participated and self-rated their anxiety state, sleep quality, and mental health (i.e., psychological, emotional, and social) during the COVID-19 pandemic. Six participants were excluded from the study due to mismatched age, gender, and/or medical expertize to the screening phase taken four years earlier. The 91 valid participants were comprised of 27 physicians treating COVID-19 patients on a daily base, for at least 2 months (COVID-19 group), and 64 physicians that are routinely working as senior physicians in the hospital departments (non-COVID-19 group).

Altogether, the screening phase tested two physiological characteristics of the physicians. Four years later, the physicians reported on their real-life well-being while treating COVID-19 patients. The screening physiological measures (anxiety and attention) were tested as predictors of the upcoming reported measures (generalized anxiety, well-being, and sleep quality) of the physicians.

**Auditory Sustained Attention Test (ASAT)** is a physiological attention measure based on an automated human startle response monitoring system (SR-HLAB™, San Diego Instruments) used to deliver acoustic startle stimuli and record electromyography (EMG) activity. First, skin was cleaned with an abrasive skin cleaning gel (LEMONPREP™, Mavidon, NC, USA) to remove dead skin cells. Two electrodes (4 mm recording area, EL254, Biopac systems Inc., CA, USA) attached to adhesive disk (ADD 204, Biopac systems Inc., CA, USA) filled with SIGNAGEL® (Parker labs, NJ, USA) and were placed below the pupil on the orbicularis oculi muscle and a third reference electrode was placed on the mastoid bone. Data were recorded at 1 Khz sampling rate with a Band-Pass Filter of 10–300 Hz. For each trial, data analysis was performed on the first 300 msec time window including the EMG maximal peak. Each session started with a 3-minute acclimatization period with a 60 dB background noise level, delivered continuously throughout the session. Next, 26 trials were delivered and included eight randomly delivered trials of a single 30 ms 114 dB “pulse alone” startle stimulus to evaluate individual startle response (i.e., anxiety level), two “pre” stimuli trials (a single 86 dB pulse), and eight “pre-pulse” trials that consist of a single 114 dB pulse preceded (120 ms inter-stimulus-interval) by a 20 ms pre-pulse of 26 dB above background noise (i.e., 86 dB). Additional eight trials with “no stimulus” delivered to monitor for baseline EMG activity and noise levels. ASAT was calculated as the percentage of response inhibition: 100-(max response to “pre-pulse” trial/max response to “pulse alone” trial × 100)^13^ to reflect attention vigilance. The stimuli delivery, recording and analysis were carried out using the Mindtension software (Mindtension, Israel).

**The Generalized Anxiety Disorder 7-Items Scale (GAD-7)** is a self-report questionnaire with seven statements describing symptoms of generalized anxiety. Subjects were instructed to determine the frequency they are disturbed by the problem in each statement during the last 2 weeks, on a Likert scale from 0-not at all to 3-almost every day. Scores over 10 and over 15 are considered as a risk and high-risk for developing an anxiety disorder, respectively. The GAD-7 has validity and internal consistency with a reliability coefficient (Cronbach’s alpha) of 0.89^23^.

**The Mental Health Continuum-Short Form (MHC-SF)** is a questionnaire used to assess three aspects of well-being (psychological, emotional, and social)^24^. This questionnaire consists of 14 items representing the construct definition of well-being for each facet (Cronbach’s alpha = 0.89)^25^.

**The Pittsburgh Sleep Quality Index (PSQI)** is a questionnaire (Cronbach’s alpha = 0.83)^26^ used to quantify the patterns and quality of sleep. The subjects were instructed to self-rate the following sleep domains: sleep duration, sleep disturbances, sleep onset latency, daytime dysfunction, habitual sleep efficiency, sleep quality, and use of sleep medication over the last month, into the pandemic. Answers scoring was based on a 0–3 Likert scale, whereby a global sum of 5 or more indicates poor sleep.

## Cluster analysis

Cluster analysis is an ML iterative partitioning method for identifying similarities among individuals and partitions the sample accordingly^27,28^ (i.e., divide data into subgroups of individuals with high heterogeneity). The groupings are constructed such that the degree of association is strong between members of the same cluster and weak between members of different clusters^29^. We performed Cluster analyses using the K-means algorithm, a common method to analyze clusters^30^, with a priori specification of two clusters.

We tested the following properties: (A) the physiological measures of startle (anxiety) and ASAT (attention) impairments; (B) anxiety measured by startle response (screening) and GAD-7 report (test phase); (C) mental health psychological, social and emotional aspects; (D) sleep duration report and startle impairment. The sensitivity, specificity, and accuracy of the prediction are depicted for each analysis. Asterisk represents the real-life group and circle represents the algorithm prediction.

### Statistical analysis

A Two-way ANOVA for mixed design with group (COVID-19 or non-COVID-19) as between-subject factor and test time as within-subject factor was performed, followed by two-sided student’s *t* test as a post hoc test or for the univariate difference between two groups. Equal variances were assumed and examined by Levin’s test. In case of dissimilar variances between the groups, the number of df was reduced accordingly. For all tests, we added effect size calculations (i.e., partial *η*^2^ for ANOVA and Cohen’s or Hedges’g for two groups comparisons when the samples are equal or unequal, respectively). When the dependent variables were categorical (i.e., PSQI) we used Mann–Whitney *U* test. Results were considered significant if *P* value < 0.05. Results are displayed as mean ± SEM, unless otherwise specified. To predict the self-reports by the screening phase physiological attention and anxiety measures, we utilized a forward hierarchical linear regression.

Physiological measures and self-reports were analyzed using *k*-means clustering on the dataset, an iterative ML technique, which organizes the physicians into two clusters based on similarity. *K*-means cluster was performed by testing 20 iterations with 50 attempts. A comparison between the actual measurements and the prediction was calculated to provide sensitivity, specificity, and accuracy measures. Owing to age difference between the groups, it was accounted as a covariate.

### Ethics statement

All procedures contributing to this work comply with the ethical standards of the Helsinki Declaration, as revised in 2008. All procedures were approved by the Emek Medical Center (EMC-080-12 and EMC-0158-16), as well as the Technion’s (91-2020) institutional review board.

### Consent statement

Verbal consent was witnessed and formally recorded as written consent to be fully anonymized and not to be identified via the manuscript.

## Results

### Demographics, sleep duration, and nicotine and caffeine intake

COVID-19 physicians were significantly younger (36.44 years; 95% CI, 33.88–39.00) than the non-COVID-19 physicians (40.78 years; 95% CI, 37.94–42.92; *t*_(57)_ = 2.277, *P* < 0.027), consumed more caffeine (95% CI, 7.17–10.30) than the non-COVID-19 group (95% CI, 2.72–5.46; *t*_(103)_ = 6.276, *P* < 0.001), and tended to smoke more cigarettes during the shift (95% CI, 0.50–6.30) compared with non-COVID-19; 95% CI, 0.35–1.77; *t*_(27)_ = 1.945, *P* > 0.062), as summarized in Table 1. The COVID-19 group is comprised of 20 internal medicine physicians (one is also a psychiatrist) and seven physicians working in emergency departments, all volunteered to move from their regular wards to specific COVID-19 wards. The non-COVID group is comprised of 31 internal medicine, 16 physicians working in emergency departments, 8 psychiatrists, 16 gynecologists, and 7 surgeons.

**Table 1:** Demographics and baseline characteristics.

	COVID-19 (*n* = 27)	Non-COVID-19 (*n* = 78)	Test	*P*
Males	19	40	*X*^2^_(1)_ = 2.969	0.085
Females	8	38		
Age (years)	36.44 (±6.47)	40.78 (±6.29)	*t*_(57)_ = 2.277	0.027
Nicotine consumption Mean (SD), cigarettes	3.4 (±2.19)	0.62 (±1.18)	*t*_(103)_ = 1.945	0.062
Caffeine consumption Mean (SD), cups	8.74 (±3.95)	3.44 (±2.26)	*t*_(103)_ = 6.276	0.0001

Sex and age distribution of COVID-19 and non-COVID-19 physicians, as well as 24-hrs nicotine (average number of cigarettes) and caffeine consumption.

### Anxiety physiological measure

**Fig. 1:**
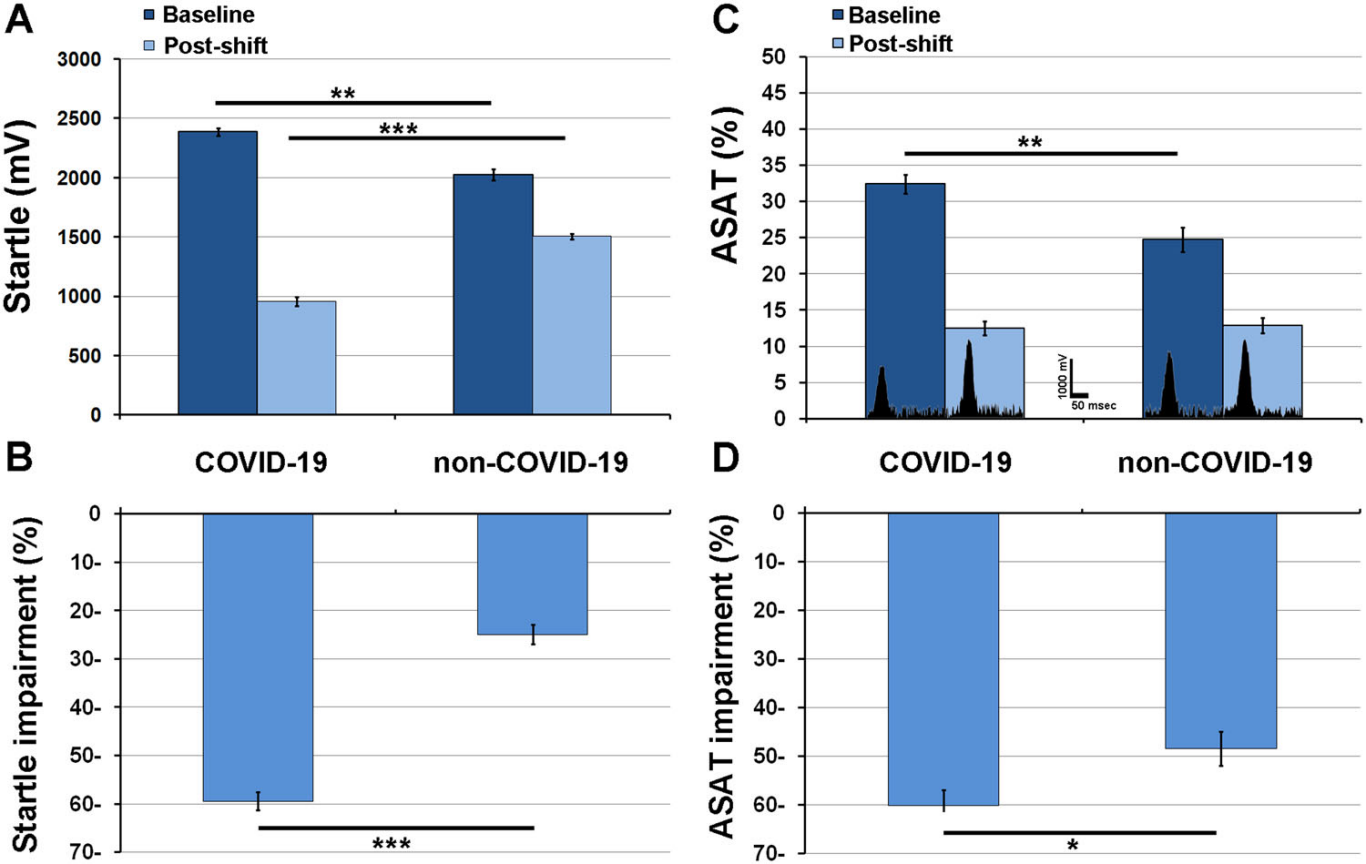
COVID-19 physicians showed significantly larger startle and attention vigilance compared with non-COVID-19 physicians. **A** Baseline startle response followed by post-shift response in COVID-19 versus non-COVID-19 group. **B** The % impairment (100 × (post-shift—baseline)/baseline) reflects the magnitude of impairment observed in the COVID-19 group, compared with non-COVID-19 group. **P < 0.01, ***P < 0.0001 (two-sided *t* test). **C** Baseline and post-shift attention levels of COVID-19 compared with non-COVID-19 physicians. Representative EMG responses illustrate the inverse relationship between the inhibited response and the ASAT performance. **D** The % attention impairment (100 × (post-shift—baseline)/baseline) reflects the magnitude of impairment observed in the COVID-19 group, compared with the non-COVID-19 group. *P < 0.05, **P < 0.01 (two-sided *t*-test).

Two-way ANOVA for mixed design revealed no significant effect between groups (*F*_(1,103)_ = 2.094, *P* > 0.153), however, we found a significant effect for test time (*F*_(1,103)_ = 15.979, *P* < 1 × 10^−7^, *η*^2^ = 0.205), as well as for the interaction group × time (*F*_(1,103)_ = 7.598, *P* < 8 × 10^−4^, *η*^2^ = 0.109). Post hoc independent samples *t* test showed significant differences (Fig. 1A) at baseline levels between groups (95% CI: COVID-19: 2317.69–2453. In all, compared with non-COVID-19: 1982.32–2068.46; *P* < 0.001) as well as post 24 hours shift challenge (95% CI: COVID-19: 883.14–1029.49 compared with non-COVID-19: 1482.31–1529.61; *P* < 0.0001). The COVID-19 group had a sizable and significant deterioration (Fig. 1B) in startle levels (95% CI: −63.18 to −55.73) compared with the non-COVID-19 group (95% CI: −26.97 to −22.99) *P* < 0.0001), following a 24-hrs shift challenge.

### Attention physiological measure

Similar to the startle response results, measuring ASAT, Two-way ANOVA for mixed design revealed no significant effect between groups (*F*_(1,103)_ < 1), however, we found a significant effect for test time (*F*_(1,103)_ = 6.802, *P* < 1 × 10^−2^, *η*^2^ = 0.099), with no interaction group × time (*F*_(1,103)_ < 1). Post hoc independent samples *t* test showed significant differences in ASAT (Fig. 1C) at baseline levels between groups (95% CI: COVID-19: 29.85–34.97 compared with non-COVID-19: 22.84–26.61; *P* < 0.001). Moreover, COVID-19 group had significantly larger deterioration (95% CI: −66.64 to −53.66; Fig. 1D) in attention level (i.e., % ASAT impairment) compared with the non-COVID-19 group (95% CI: −55.74 to −43.15; *P* < 0.043).

### The GAD-7

**Fig. 2:**
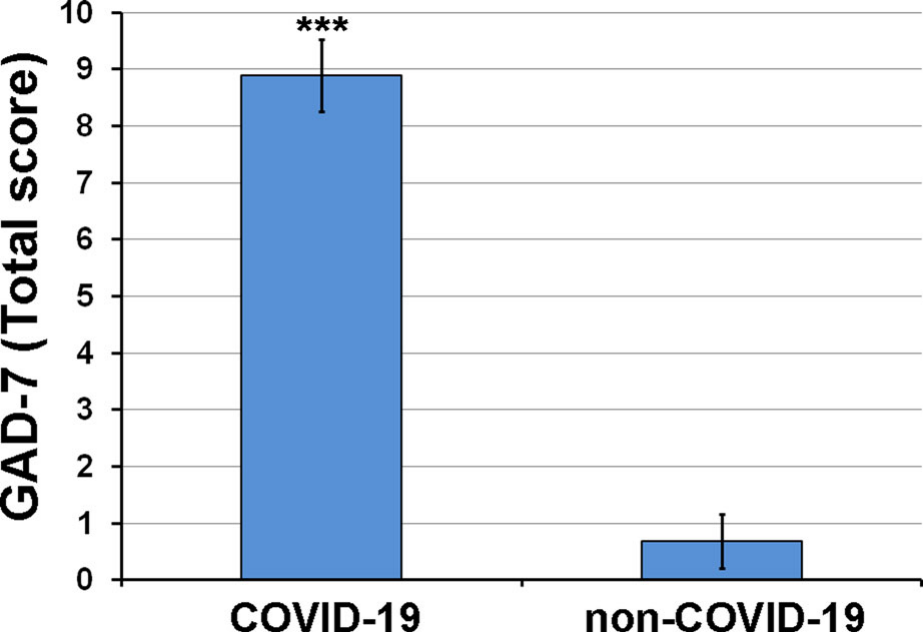
COVID-19 physicians reported significantly higher anxiety scores compared with non-COVID-19 group. The Generalized Anxiety Disorder 7-Items Scale (GAD-7) in COVID-19 physician**s** compared with non-COVID-19 group. ***P < 0.0001 (two-sided *t* test).

GAD-7 total scores among the COVID-19 group were significantly higher (95% CI: 7.62–10.14; Fig. 2) compared with the non-COVID-19 group (95% CI: 0.29–1.10; *t*_(32)_ = 6.84, *P* < 4.3 × 10^−2^). Importantly, a GAD-7 total score of above 10 is considered as a risk of developing an anxiety disorder. Six out of 27 physicians (22%) of the COVID-19 group scored above 10 versus not a single subject in the non-COVID-19 group.

### The MHC-SF

**Fig. 3:**
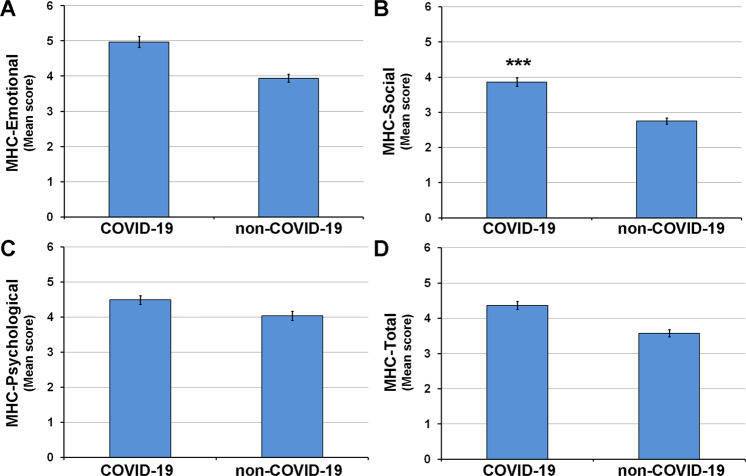
COVID-19 physicians reported significantly higher score in the social MHC-SF well-being dimension. The Mental Health Continuum-Short Form (MHC-SF) in COVID-19 physicians compared with non-COVID-19 group: **A** emotional aspect, **B** social aspect, **C** psychological aspect, **D** MHC-SF total scores. ***P < 0.0001 (two-sided *t* test).

Evaluating well-being aspects among physicians during the pandemic showed higher scores in the COVID-19 group compared with the non-COVID-19 (Fig. 3). Independent samples *t* test showed a significant difference in the general score of social aspect (95% CI: COVID-19: 4.12–4.60 compared with non-COVID-19: 3.44–3.72; *t*_(40)_ = 2.867, *P* < 7 × 10^−7^). Specifically, physicians treating COVID-19 patients reported that they had something important to contribute to society (*P* < 0.015), felt stronger relationship to the community (*P* < 0.0001), had higher scores for the statement “people are basically good” (*P* < 0.0001), and higher scores for the statement “the way how our society works makes sense to you” (*P* < 0.0001). Notably, the COVID-19 group had higher non-statistically significant emotional and psychological well-being scores.

### The PSQI

The COVID-19 group had stronger changes in the patterns and quality of sleep compared with the non-COVID-19 group (Supplementary Table 1). Specifically, the COVID-19 group had longer sleep duration (95% CI: COVID-19: 1.44–1.94 compared with non-COVID-19: 0.15–0.37; *U*_(27,64)_ = 313.00, *P* < 0.0001), longer sleep onset latency (95% CI: COVID-19: 1.75–2.01 compared with non-COVID-19: 0.68–0.94; *U*_(27,64)_ = 326.50, *P* < 0.0001), deteriorated daily function (95% CI: COVID-19: 2.54–2.91 compared with non-COVID-19: 1.28–1.60; *U*_(27,64)_ = 143.00, *P* < 0.0001), poorer sleep quality (95% CI: COVID-19: 2.75–3.01 compared with non-COVID-19: 1.74–2.10; *U*_(27,64)_ = 258.00, *P* < 0.0001), and worse global PSQI score (95% CI: COVID-19: 9.97–11.25 compared with non-COVID-19: 4.57–5.27; *t*_(89)_ = 9.751, *P* < 0.0001).

### Prediction of COVID-19 mental health using physiological screening of attention and anxiety

We used forward hierarchical regression to predict self-reported mental health outcomes from the physiological parameter to. First, we found that both group and baseline startle response significantly predict anxiety score measured by GAD-7 (*F*_(2,102)_ = 89.01, *P* < 1 × 10^−7^) explaining *R*^2^ = 63.6% of the variance (GAD-7 total score = 6.685 − 4.41 × group − 0.108 × baseline startle response).

Next, we found that group, post 24 hours ASAT and post 24 hours startle response predicted the emotional aspect of MHC-SF (*F*_(3,87)_ = 11.41, *P* < 1 × 10^−4^), explaining *R*^2^ = 28.2% of the variance (MHC-SF emotional score = 4.14 + 0.913 × group + 0.78 × post 24 hours ASAT—0.002 × post 24 hours startle response).

We found that group, post 24 hours startle and ASAT impairment were significant predictors for PSQI total score (*F*_(3,87)_ = 207.71, *P* < 1 × 10^−7^) explaining *R*^2^ = 78.6% of the variance (PSQI total score = 18.87−3.073 × group−0.006 × post 24 hours startle response−0.013 ASAT impairment).

Finally, following the above-reported parametric differences and regression prediction models, we examined whether ML could distinguish between the COVID-19 and the non-COVID-19 groups.

**Fig. 4:**
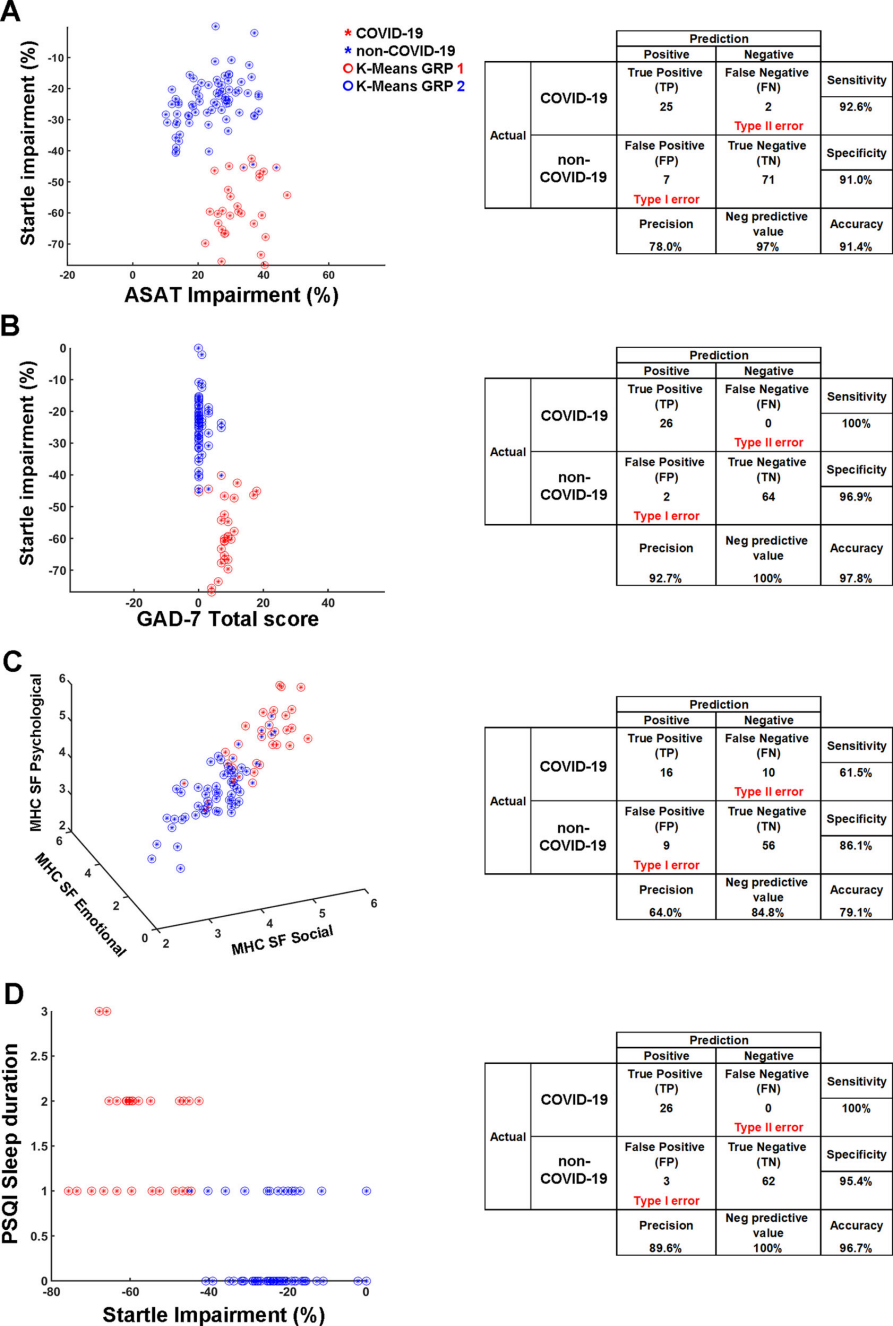
Comparing clustering of COVID-19 and non-COVID-19 groups. Clustering of the differences between COVID-19 and non-COVID-19 physicians. **A** The physiological measures of startle (anxiety) and ASAT (attention) impairments, **B** Anxiety measured both by startle response (while residents) and GAD-7 report (during the COVID-19 pandemic). **C** Mental health psychological, social, and emotional aspects; and **D** sleep duration report and startle impairment. Sensitivity, specificity, and accuracy of the prediction are depicted for each analysis. The asterisk represents the real-life group and the circle represents the algorithm prediction.

Figure 4 shows a clear distinction between the subgroups with good correlation, sensitivity, and specificity between the real-life group and the algorithm predictions.

## Discussion

Both physicians treating COVID-19 and non-COVID-19 patients showed signs of abrasive routine work. However, in the screening phase, in a retrospect, the COVID-19 group manifested higher anxiety and attention vigilance, and in the current test phase, they reported higher social support, higher anxiety score, and low quality of sleep.

The pattern of symptoms comprised of high anxiety and attention vigilance together with low quality of sleep—all under chronic stress, is suggested to exacerbate the emergence of post-traumatic stress symptoms^31^. In PTSD, survival mechanisms are dominant during a stressful situation and characterized by a transitional state of heightened arousal and hypervigilance, aimed at coping with an immediate threat^32^. This state resembles, exempli gratia, one of the soldiers that need to fully operate during combat.

Twenty-four hours shift challenge revealed important information regarding the modulation of anxiety and attention vigilance (i.e., % impairment). Retrospective analysis of these results recorded during the physicians’ residency (4 years prior to the COVID-19 pandemic) indicated significant impairment of the COVID-19 group’s attention vigilance following extended 24 hours shift challenge. Notably, we further examined whether the objective physiological pre-COVID-19 measures and self-reports may be organized through the ML tool to assess the multidimensional signature of the physicians’ mental health. Indeed, the results indicated a clear and strong prediction of the real-life group pertinence by the algorithm, with overall high sensitivity and specificity, supporting the multidimensional characteristics of the COVID-19 or non-COVID-19 groups.

The COVID-19 pandemic has created a sudden and novel stressor across medical staff^33^, especially under rapidly changing official health guidelines that augmented uncertainty. Thus, emphasizing the importance of health support and risk-assessment of medics at the COVID-19 frontline. The similarity to PTSD triggers, support the immediate need to monitor the susceptibility to develop PTSD, among physicians treating COVID-19 patients. Dysregulated anxiety and attention vigilance may reflect individual differences in top–down attentional control, which influence the expression of attentional bias such as in PTSD^34^.

The ML tool unequivocally supported the real-life data by high correlations with the algorithm predictions, accompanied by high sensitivity, specificity, and accuracy of the predictions. The ability to identify and integrate risk indicators makes this a promising method for estimating a probabilistic risk of post-traumatic stress psychopathology based on biological, psychological, and social information^19^.

This integrative approach is particularly crucial for evidence-based implementation of clear guideline for the worldwide health authorities to monitor and support the reduction of physicians’ anxiety during routine workload, especially during the pandemic. Thus, the current results offer a practical and physiological strategy rather than a subjective, questionnaire-based one. Implementation of preventative therapeutic emotional support system to medical staff during uncertain stressful times, such as routine professional CBT-based group meetings^35^, may be beneficial for those at risk of developing post-traumatic symptomatology. Further, ASAT scores of all physicians in the hospital may be useful routinely as well as in future crises by predicting those who are at risk.

In summary, supported by our pre-COVID-19 physiological measures and self-reported COVID-19 data, the results correlate between the anxiety level of medical residents and their self-reported anxiety while becoming senior physicians. Moreover, the ML findings pointed out the need for guide-directed interventions aimed at those susceptible to develop PTSD owing to the consequences of fighting at the forefront of the COVID-19. Thus, highlighting the importance of implementing mental health interventions into care systems, also outside the scope of the current pandemic.

## Supplementary information

Supplementary Table 1
