# Supplementary material for: Physiological parameters of mental health predict the emergence of post-traumatic stress symptoms in physicians treating COVID-19 patients
Source: Transl Psychiatry. 2021 Mar 15;11:169. doi: 10.1038/s41398-021-01299-6 (PMC7957277; doi:10.1038/s41398-021-01299-6)
Supplement: Supplementary file 1 — Supplementary Table 1 [file 41398_2021_1299_MOESM1_ESM.docx]

Supplemental material

**Supplementary Table 1. The Pittsburgh Sleep Quality Index (PSQI): Quantifying the patterns and quality of sleep.** All measures were analyzed by Mann-Whitney U test, except Global PSQI score that was analyzed by Two-sided student’s t-test.

|  | **COVID-19**  **(n=27)** | **Non-COVID-19**  **(n=78)** | ***P*** |
| --- | --- | --- | --- |
| **Sleep Duration Mean (SD), score** | 1.69  (± 0.61) | 0.27  (± 0.35) | 2x10^-6^ |
| **Sleep Disturbance Mean (SD), score** | 1.00  (± 0) | 0.52  (± 0.5) | n.s |
| **Sleep Latency Mean (SD), minutes** | 1.88  (± 0.32) | 0.80  (± 0.47) | 1x10^-7^ |
| **Daily Functioning Mean (SD), score** | 2.73  (±0.45) | 1.45  (± 0.59) | 1x10^-7^ |
| **Sleep Efficacy Mean (SD), score** | 0.42  (± 0.7) | 0.00  (± 0) | 5x10^-3^ |
| **Sleep Quality Mean (SD), %** | 2.88  (± 0.32) | 1.88  (± 0.45) | 1x10^-7^ |
| **Sleep Medication Use No.** | 0.00 | 0.00 | n.s |
| **Global PSQI Mean (SD), score** | 10.62  (± 1.57) | 4.92  (± 1.34) | 1x10^-7^ |
